# Prodigious submarine landslides during the inception and early growth of volcanic islands

**DOI:** 10.1038/s41467-017-02100-3

**Published:** 2017-12-12

**Authors:** James E. Hunt, Ian Jarvis

**Affiliations:** 1 National Oceanography Centre, Waterfront Campus, University of Southampton, European Way, Southampton, Hampshire SO14 3ZH UK; 2 Department of Geography and Geology, Kingston University London, Penrhyn Road, Kingston upon Thames, KT1 1LQ UK

## Abstract

Volcanic island inception applies large stresses as the ocean crust domes in response to magma ascension and is loaded by eruption of lavas. There is currently limited information on when volcanic islands are initiated on the seafloor, and no information regarding the seafloor instabilities island inception may cause. The deep sea Madeira Abyssal Plain contains a 43 million year history of turbidites among which many originate from mass movements in the Canary Islands. Here, we investigate the composition and timing of a distinctive group of turbidites that we suggest represent a new unique record of large-volume submarine landslides triggered during the inception, submarine shield growth, and final subaerial emergence of the Canary Islands. These slides are predominantly multi-stage and yet represent among the largest mass movements on the Earth’s surface up to three or more-times larger than subaerial Canary Islands flank collapses. Thus whilst these deposits provide invaluable information on ocean island geodynamics they also represent a significant, and as yet unaccounted, marine geohazard.

## Abstract (web summary)

As volcanic islands grow via subaerial shield-building and edifice growth, large submarine landslides occur. Here, the authors reconstruct the emergence of the Canary Islands via turbidites created by submarine landslides as the islands grew, showing that slides are multi-stage.

## Introduction

**Fig. 1:**
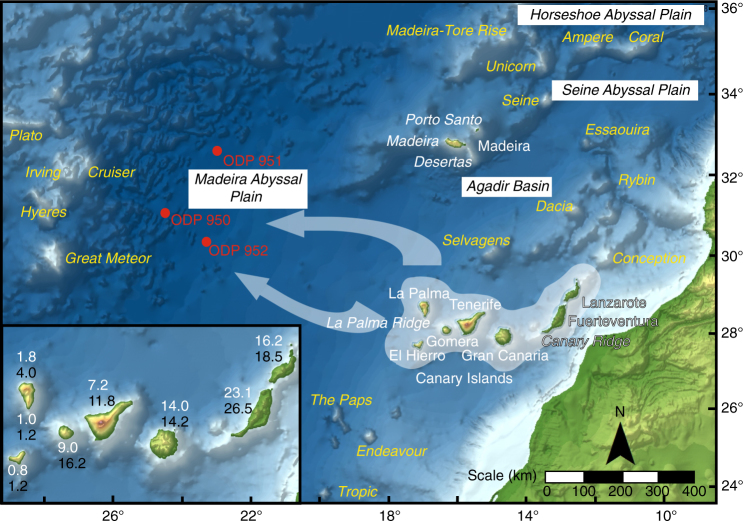
Map of the Canary Basin on the Northwest African Passive Margin. Featured are the Canary and Madeira archipelagos, with an indication of topography and deep sea bathymetry sourced from GEBCO data. Also shown are the positions of the ODP Leg 157 Sites in the Madeira Abyssal Plain. Halo area surrounding the Canary Islands represents the potential source area of the landslides and their associated turbidites studied here. Inset is a map of the Canary Islands showing estimated ages of inception (black) and subaerial emergence (white) based on the present study. Arrows indicate the direction of turbidity currents from landslides sourced from the seafloor adjacent to the Canary Islands

Volcanic island landslides have previously been shown to primarily occur during the mature stages of subaerial shield-building and edifice growth^1,2^. During subaerial growth, the injection of dykes and effusion of both lavas and pyroclastics rapidly over-steepen, load and ultimately cause flank instability^1,3–5^. The resulting flank collapses are often prodigious in volume, with many past slides in the Hawaiian and Canarian archipelagos exceeding 100 km^3^, which far exceed terrestrial slides by several orders of magnitude^2,6–9^. In the Canary archipelago, landslides during subaerial island growth are recorded by large scallop-shaped escarpments, debris avalanche deposits on the proximal submarine island flanks, and large-volume volcaniclastic turbidites in adjacent deep-sea depocentres (Fig. 1)^2,8–11^. While the volumes of these volcaniclastic turbidites represent only a proportion of the original slide mass, they still record the timing of the slide and are indicative of the scale of the failure^2,9^. Indeed, the turbidite record of the Madeira Abyssal Plain has been shown to excellently record the history of subaerial volcanic island flank collapses, for example all eight of the subaerial flank collapses in the Western Canary Islands in the last 1.5 Myr are recorded^9^. However, there are some notable caveats, such that slides from Tenerife (Icod, Orotava, Guimar and Roques de Garcia) and El Hierro (El Hierro, El Julan and Tinor) show excellent chronological agreement with onshore age estimates, while the landslide history from these turbidites identifies a La Palma-sourced landslide dated at 485 ± 10 ka at least 35 ka younger than its onshore contemporary^8,9,12–14^. An extended 17 Myr turbidite record from the Madeira Abyssal Plain has also been shown to accurately record past subaerial collapses further back in time^2^. It must be acknowledged that not all slides necessarily generate turbidity currents. While small-volume failures may not reach the distal Madeira Abyssal Plain, those failures of significant large-volume (>10 km^3^) are shown to regularly reach the basin, thus representing a potentially unbiased record of landslide history^2,9^. The excessive volumes and potentially catastrophic nature of these volcanic island landslides necessitate characterising past events in order to better understand present hazards and levels of risk associated with them^15^.

Little is known about the early seamount stages of volcanic island growth during initial inception and later emergence, what inherent internal stresses may be exerted on growing island flanks during this stage, and whether prodigious seafloor mass wasting may result. The early seamount stage of island growth is suggested to involve the development of large tensional stresses associated with crustal doming and fracturing caused by magma ascent and rift development^1,3–5^. Initial emplacement of the island on the seafloor and its subsequent subaerial emergence represents the largest relative emplacement of volcanic mass on the ocean crust during the life cycle of an island. Seamount growth leading to island inception also involves the emplacement of surficial, low-porosity, and structurally weak hyaloclastites that both substantially increase slope angles and introduce interbedded low-permeability materials that facilitate failure^16^.

Submarine landslides have been identified on the flanks of submarine seamounts associated with mid-ocean ridges^16^. Such seamounts may take 30–60 Myr to develop to over 2,000 m seafloor elevations by relatively gradual addition of volcanic materials^17^. Submarine landslides have also been recognised affecting volcanic seamounts elsewhere (e.g., Henderson Seamount, offshore Baja^18^, Moua Pihaa, Society^19^, and MacDonald and Arago Seamounts, Austral^19^). Landslides, proximal debris-avalanches and their often associated turbidites reflecting collapse of subaerial-to-proximal submarine volcanic island slopes have been identified around seamounts and emergent volcanic islands (e.g., the Canary Islands^2,8,9,11,20^, Hawaiian Islands^6,7,21^, Reunion Island^22,23^, Pico Island, Azores^24^, Tristan Da Cunha Island^25^, Gough Island^26^, Guadalupe Island^16^, and Polynesian Islands^19^). These particular landslides often form lobate deposits adjacent to the islands that comprise volcanoclastic materials sourced from volcanic edifice. Here, we propose a distinct group of turbidites in the Madeira Abyssal Plain over the last 43 Myr represent large-volume failures of seafloor immediately adjacent to volcanic islands that instead comprise accumulated hemipelagite sediments and distributed volcaniclastic sediments beyond the volcanic island slopes. We propose that these landslides and the resultant turbidity currents occurred in response to magma ascension and doming of the seafloor adjacent to the volcanic island (e.g., source area delineated in Fig. 1) associated with inception, ascension and emergence during the seamount-phase of growth and later subaerial-phase of growth. Volcanic islands above intra-plate plumes grow more rapidly during their seamount-phase their contemporaries and become subaerial volcanic edifices in an order of magnitude less time than is taken to develop mid-ocean ridge seamounts. For example, El Hierro or La Palma in the Canary Islands have taken < 1.2 Myr to develop to major subaerial edifices (Fig. 1)^27–29^. This rapid growth of volcanic islands may substantially increase the risk of flank instabilities. Despite the large emplacement of volcanic mass upon the oceanic crust, the deposition of surficial materials potentially prone to failure and the rapidity of growth, there is currently no record of seafloor instability during the early seamount-phase of volcanic island growth nor prior to major periods of island building.

Here, we present the first record of submarine landslides during the inception and early growth of volcanic islands sourced from failure of accumulated hemipelagite and volcaniclastic debris on their submarine slopes and adjacent seafloor (see Fig. 1). We further show that these landslides are among the largest mass movements on Earth’s surface. We suggest that this is because doming of the seafloor occurs in response to magma ascension, and subsequently also occurs prior to major periods of subaerial volcanism. We theorise that pale-grey ‘non-volcanic’ turbidites deposited on the Madeira Abyssal Plain, offshore NW Africa, represent seafloor failures during the inception, seamount growth, emergence and later subaerial edifice growth of the adjacent Canary Islands. An unprecedented long turbidite sequence from ODP Sites on the Madeira Abyssal Plain (Sites 950, 951 and 952) has previously revealed a 17.0 Ma record of submarine landslides offshore NW Africa, including subaerial landslides from the Canary Islands and adjacent continental margin^2,30–32^. The volumes (>10 km^3^), runouts (>1,000 km) and spatial distributions (>150,000 km^2^) of these sediments indicate a source from a submarine landslide, and negate possible alternative sources from pyroclastic flows, hypopycnal flows or river delta failures that have much smaller volumes and distributions^2,9^. Our study investigates the origin and magnitude of the pale-grey ‘non-volcanic’ turbidites from this 17.0 Ma record and a new additional 18.0–43.0 Ma record from ODP Site 950. We also investigate the prevalence of multi-stage collapse as a failure mechanism for these slides. The aim is to test whether these voluminous deposits represent submarine flank collapses that occur during inception and emergence stages of volcanic island growth in the Canary Islands, and whether they pose a novel and significant geohazard.

## Results

### The Canary Islands and Madeira Abyssal Plain turbidites

The Canary archipelago represents an east-to-west chain of volcanic islands extending over ~500 km off the NW African passive margin (Fig. 1). The origin of this archipelago has been much debated, and competing theories exist: propagating fracture model; uplift of tectonic blocks; Canary rift model; classic plume model; blob model; and upwelling sheet model. However, a critical review by Carracedo et al.^33^ concluded that current evidence best supports growth of the islands on a ‘slow’ moving plate above a mantle plume.

Although a general east-to-west age progression is apparent in the Canary archipelago (Fig. 1), there is greater divergence along this trend when compared to the Hawaiian archipelago^1,33^. This is attributed to ‘slow’ movement (1.9 cm per year) of oceanic crust over a mantle plume and possible generation of a dual-line, compared to the single-line Hawaiian chain operating on ‘fast’ moving (10 cm per year) oceanic crust^1,33,34^. However, despite the differences in the geodynamics of the respective tectonic plates, both the Canary and Hawaiian archipelagos have had prodigious subaerial and submarine landsliding capable of generating tsunamis^35,36^.

**Fig. 2:**
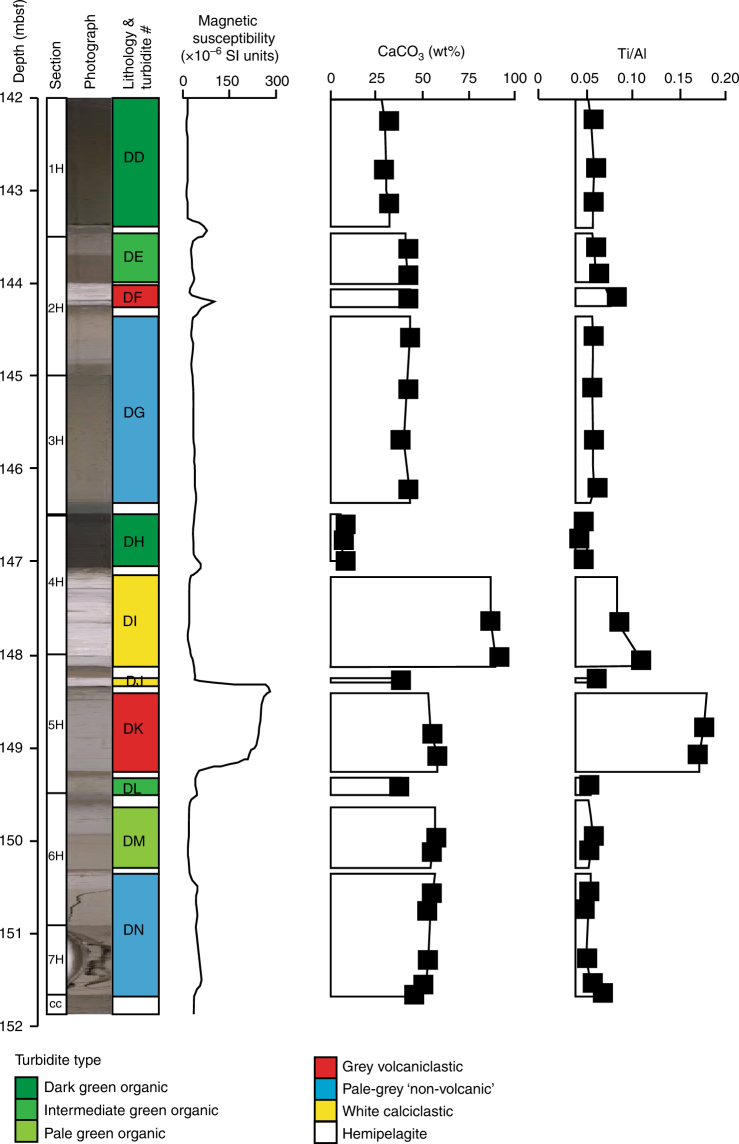
Example ODP core from Site 950 featuring sections from Core 16 H of Hole 950 at 142–152 mbsf. This illustrates all the turbidite types, with the pale-grey ‘non-volcanic’ turbidites of interest highlighted in blue (beds DG, DN). Note the pale-grey colour, but lack of magnetic susceptibility response, and low Ti/Al seen in the ‘non-volcanic’ turbidites, compared to volcaniclastic turbidite DK (red). Black squares represent location of previous samples taken for geochemical analysis

Landslides from the Canary Islands, NW African continental slope, and seamounts adjacent to the mid-ocean ridge disaggregate to form dilute turbidity currents whose deposits, known as turbidites, accumulate on the Madeira Abyssal Plain in water depths of greater than 5,000 m (Figs. 1 and 2). The stratigraphy recovered from the Madeira Abyssal Plain at ODP Sites 950, 951 and 952 represents an unequivocal continuous archive of landslides over the past 17.0 Ma^2,31,32^, which we extend here to 43.0 Ma using data from Site 950. Previous work has identified green organic-rich turbidites sourced from the NW African continental slopes, grey volcaniclastic turbidites sourced from the subaerial Canary Islands, and white calciclastic turbidites sourced from local seamounts and ridges (Fig. 2)^10,30,31,37^. There are also metre-thick pale-grey ‘non-volcanic’ turbidites in the Madeira Abyssal Plain stratigraphy at ODP Sites 950, 951 and 952, but neither have the origin, magnitude nor timing of these beds, until now, been identified (Fig. 2)^2,30^. The term ‘non-volcanic’ was used previously to distinguish these pale-grey beds from the grey volcaniclastic turbidites that represent subaerial flank failures of the Canary Islands^2,30^. We hypothesise that these pale-grey ‘non-volcanic’ beds represent failure of seafloor and submarine flanks of the Canary Islands during inception and early island growth. The larger than 10 km^3^ volume (commonly > 100 km^3^) negates origins from sources other than large submarine landslides^2,9^.

### Provenance of the pale-grey non-volcanic turbidites

The pale-grey ‘non-volcanic’ turbidites of this study are pale-grey, decimetre to metre-thick, laminated silts and muds that represent the fine-grained Bouma Td and Te divisions of large dilute turbidity currents sourced from submarine landslides (Fig. 2). The sharp-bases that are laminated and the fining-upwards grain-size with weakly bioturbated tops set these deposits distinctively apart from the heavily bioturbated white to dark brown/red hemipelagites (background sediment) in which they are encased (Fig. 2). They are also distinctly different from the grey, coarser-grained volcaniclastic turbidites.

**Fig. 3:**
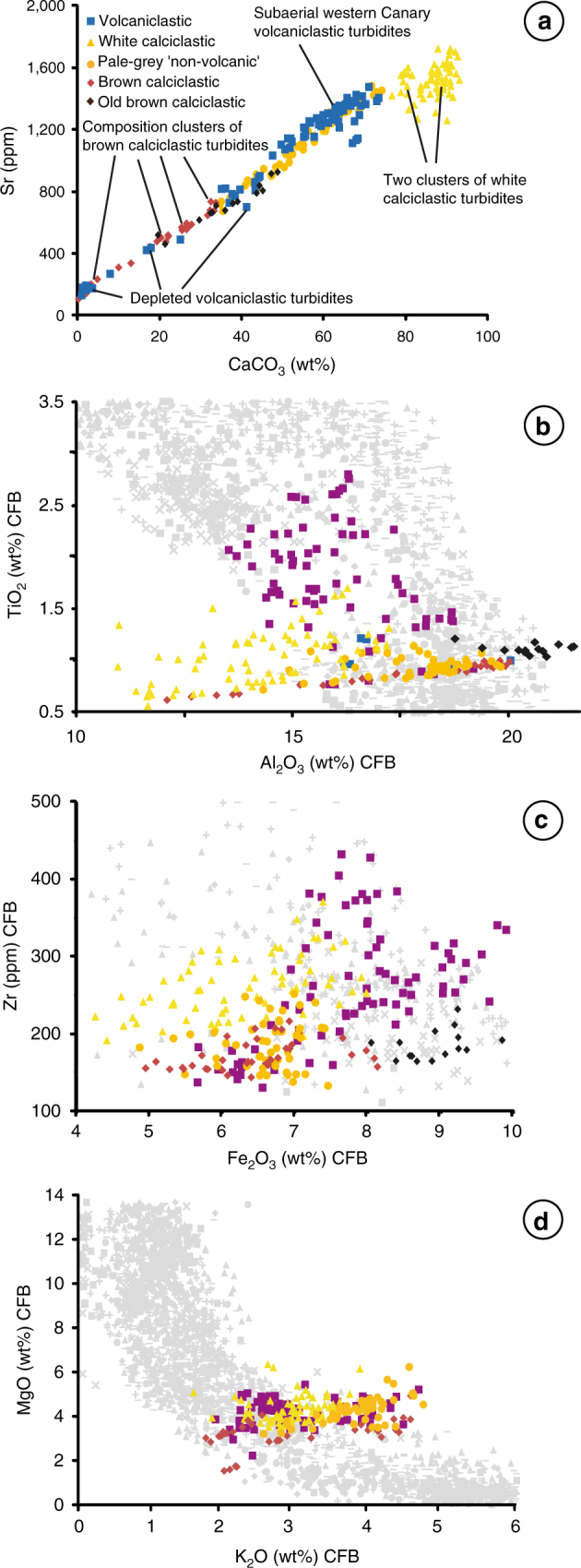
Variation plots of mudcap geochemical compositions of the volcanic pale-grey non-volcanic and calciclastic turbidites at Site 950. **a** Sr against CaCO_3_. Data calculated on a carbonate-free basis (CFB) featuring: **b** TiO_2_ against Al_2_O_3_; **c** K_2_O against Al_2_O_3_; and **d** Zr against total iron (Fe_2_O_3_). Data of turbidite compositions overlain on the composition of terrestrial volcanics from the Canary Islands (grey) and Madeira (black); volcanic data sourced from the GEOROC database at Max-Plank Institut für Chemie (http://georoc.mpch-mainz.gwdg.de/georoc). Reliability of major element oxides > 2 wt% present standard deviations 0.7–1.5% of the value, while with values 1–2 wt% produce standard deviations 1.5–10% of the value. Accuracy of major element oxides in relation to international standard reference materials are better than 2% of the value with values > 3 wt%, while within 2–10% of the value when values 0.1–2 wt%. Reliability of trace element data have standard deviations better than 2% with values > 60 ppm, while accuracy is within 0.7–5.0% of the value with values > 60 ppm

Volcaniclastic turbidites in the Madeira Abyssal Plain represent large-volume failure of the subaerial island edifices in the Canary Islands^2,9,10,30^. They are characterised by their diagnostic dark grey colour, high magnetic susceptibility (>50 SI units) and relatively high TiO_2_ CFB (carbonate-free basis) content and high Fe_2_O_3_ CFB content (Figs. 2 and 3). White calciclastic turbidites have a diagnostic white colour, high CaCO_3_ (>78 wt%) and Sr (>1,200 ppm) contents, negligible magnetic susceptibility, and low MgO CFB, Fe_2_O_3_ CFB and Al_2_O_3_ CFB compositions (Fig. 3). These white calciclastic turbidites are thought to represent failure of carbonate-rich seafloor sediments on seamounts surrounding the Madeira Abyssal Plain draped in pelagic coccolith-rich oozes^37^.

Pale-grey ‘non-volcanic’ turbidites have high CaCO_3_, low magnetic susceptibility and low Ti/Al, which partially differentiate them from purely volcaniclastic turbidites (Figs. 2 and 3). However, mudcap geochemistry highlights that the pale-grey ‘non-volcanic’ beds have a stronger affinity to the volcaniclastic turbidites than the white calciclastic beds. The relatively high Fe_2_O_3_ CFB may also further indicate the presence of volcaniclastic materials in the flows (Fig. 3).

The origin of the pale-grey ‘non-volcanic’ turbidites is explicably linked to the Canary Islands. The geochemical composition of the sediments from these turbidites in the last 17 Myr falls within the compositional fields of the more evolved Western Canary Islands (Fig. 3). The compositions also show that Madeira or its archipelago cannot be the source (Fig. 3). Previous studies implicate the calciclastic turbidites, rich in carbonate detritus, as being likely sourced from seamounts local to the Madeira Abyssal Plain. Similarities in composition with the volcaniclastic turbidites but higher carbonate content imply that the pale-grey ‘non-volcanic’ turbidites represent failures from purely submarine regions surrounding the Canary Islands where there are mixtures of both volcaniclastic sediment and calcareous pelagites. Combined with age estimates, these changes in composition may provide further information regarding the provenance of the turbidites and the geodynamics of the islands from which they are sourced.

### Slides during island inception and early growth

**Fig. 4:**
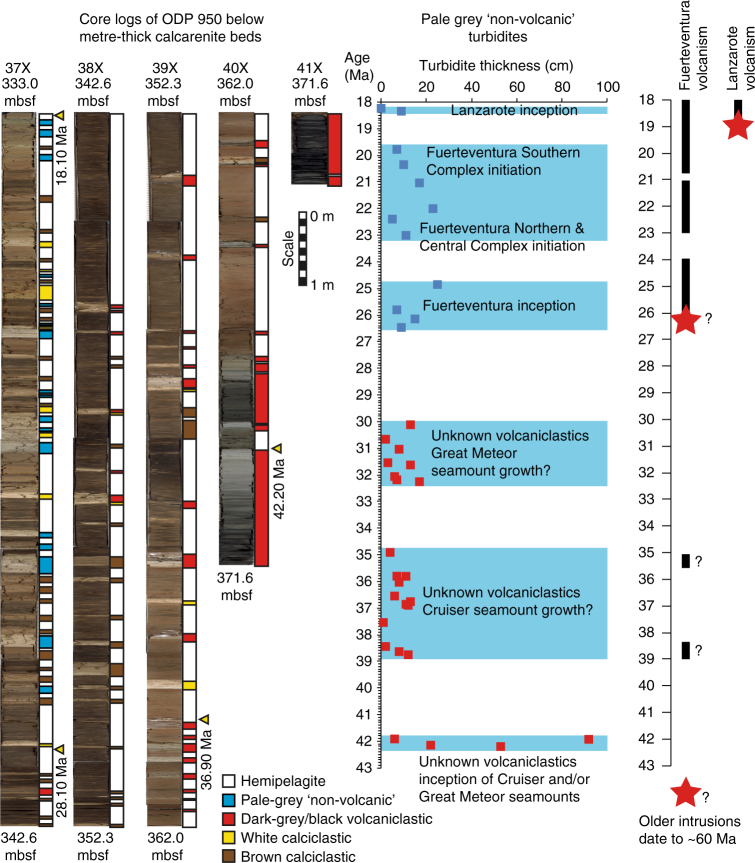
Core log of stratigraphy at ODP 950 below 330 mbsf. Temporal distribution of pale-grey ‘non-volcanic’ turbidites represented by blue lithology in legend signifying inception and emergence of the eastern Canary Islands represented by blue squares. Black layers within the oldest volcaniclastic turbidites are sand-rich with abundant basalt clasts. These black volcaniclastic turbidites represent failure of growing volcanic structures adjacent to the Madeira Abyssal Plain, and likely represent landslides from the Cruiser or Great Meteor seamounts (red squares). The ages of inception from age estimates of the Basal Complexes^29^ are show as red stars, the dates of inception of Fueteventura are compounded by dates from metamorphosed suites that have exaggerated ages

**Fig. 5:**
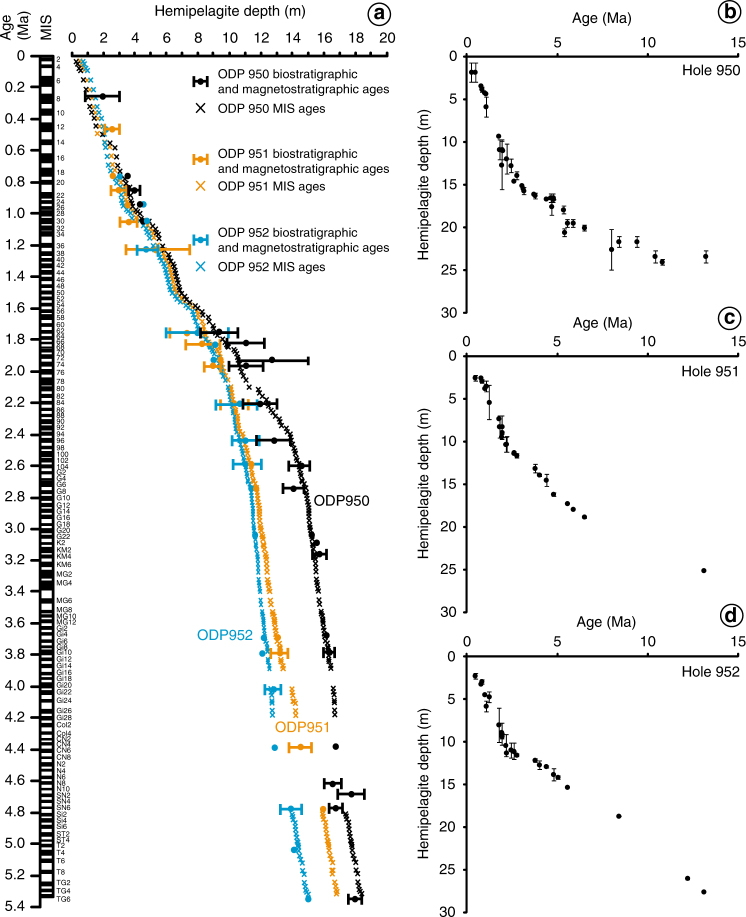
Age model for Madeira Abyssal Plain sediments. Hemipelagite depth against time **a** for the last 5.4 million years at ODP Sites 950 (black), 951 (orange) and 952 (blue) showing hemipelagites depths of coccolith first and last occurrences with error bars (circles)^38^ combined with depth and age of identified Marine Isotope Stage boundaries. Lithostratigraphy of the hemipelagite records periods of sea level change, manifested in the Marine Isotope Stage record^9,37,49,63,67^. At high sea-level there is greater calcium carbonate preservation forming white calcareous oozes younger than 2.5 Ma or pale brown to light grey hemipelagites in the older record. In contrast, dark brown clays are deposited during lowstands younger than 2.5 Ma and dark grey clays in the older record^9^. Also shown are the biostratigraphic age controls up to 15 Ma for **b** ODP Site 950, **c** ODP Site 951, and **d** ODP Site 952

The pre-7.0 Ma record of pale-grey ‘non-volcanic’ turbidites predominantly consist of thin-bedded (10–50 cm-thick) turbidites comprising silt bases and decimetre-thick mudcaps, representing the fine-grained sediment deposited out of the final suspension of turbidity currents. These are only recorded at ODP Site 951 or 950, whilst the pre-18 Ma record was only recovered at ODP Site 950 (Fig. 4). The uncertainty on the ages of the events in the 43 to 7.0 Ma record average at 5% of the given age (±0.8–2.2 Ma) based upon last and first-occurrence coccolith biostratigraphic ages^38^. In contrast, the pale-grey ‘non-volcanic’ turbidites post-dating 7.0 Ma are widely correlatable between ODP Sites 950, 951 and 952, and have much more robust age models. First, the turbidites younger than 6.5 Ma have initial age uncertainties of 1–5% of the age using coccolith biostratigraphy and magnetostratigraphy (±10–270 ka)^38^. However, this uncertainty is improved by two orders of magnitude to conservatively ±10 ka in the sediment record younger than 5.4 Ma when using the hemipelagite lithostratigraphy coupled with marine isotope stage boundaries (Fig. 5). Hemipelagite sediments record the history of sequential marine isotope stages with sharp boundaries between glacial lowstands (red-brown clays) and interglacial highstands (white carbonate oozes) of sea level that have well defined ages (Fig. 5). Almost 80% of beds younger than 5.4 Ma occur at distinct boundaries between glacial red clays and interglacial white oozes and thus can be dated conservatively to ±10 ka, those beds that lie within hemipelagite between such boundaries (20%) can be dated by interpolation to boundaries above and below to ±20 ka.

**Fig. 6:**
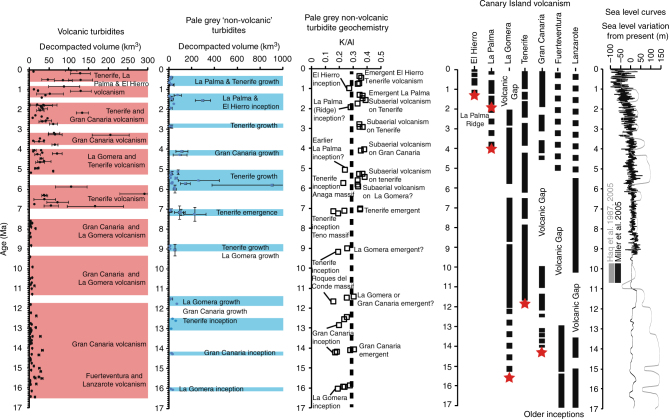
Summary diagram. Figure shows the timing and decompacted volume of pale-grey ‘non-volcanic’ turbidites (blue shaded clusters) and grey volcaniclastic turbidites (red shaded clusters). These are plotted against the temporal distribution of volcanic activity of the Canary Islands and sea level curves. Red stars indicate subaerial emergence of the islands based on terrestrial dates from basal complexes^27–30,71–75^

The turbidites in the post-7.0 Ma record represent uniform ungraded muds that range in thickness from 20 cm to 12 m, but average 2.2 m in thicknesses (Fig. 2). When these beds are decompacted they provide average depositional volumes between 100 and 200 km^3^ (Fig. 6). Turbidite FB, dated at 5.8 Ma (nomenclature from Hunt et al.^2^), represents the thickest (12 m thick) and most volumetric (900 km^3^ decompacted volume) turbidite in the Madeira Abyssal Plain. For perspective, the slide responsible for this particular bed remobilised enough sediment to cover France or the US state of Alaska in over one metre of clay and silt, equating to volumes that would take all of the Earth’s rivers over one hundred years to accumulate.

Age determination and deposit compositions have allowed the occurrence of the pale-grey ‘non-volcanic’ turbidites to be separated into specific periods. These periods broadly correlate to the oldest dates of volcanism on individual Canary Islands representing inception and later emergence and initiation of periods of major subaerial volcanic activity (Figs. 4 and 6). A distinct vertical change in composition is apparent within each group of the pale-grey ‘non-volcanic’ turbidites from <0.275 K/Al to >0.275 K/Al, which is reflected in other CFB element compositions and ratios (Fig. 6). This change may represent a transition from inclusion of more basic submarine volcaniclastic material to inclusion of more K-rich volcaniclastic material, as volcanism on the respective island matures and the respective seamount emerges subaerially. Thus, utilising compositional information of the pale-grey ‘non-volcanic’ beds (specifically K/Al < 0.275) and the turbidite ages, we can refine the age of inception of the individual Canary Islands (Fig. 6): 16.0 Ma inception of La Gomera (estimated 15.5 Ma^39,40^); 14.2 Ma inception of Gran Canaria (estimated 13.7–13.5 Ma^41,42^); 11.7–9.2 Ma inception of Tenerife Roques del Conde massif (estimated 11.8–9.6 Ma^29,43^); 7.2 Ma inception of Tenerife Teno massif (estimated 7.4–6.1 Ma^43^); 5.8 Ma inception of Tenerife Anaga massif (estimated at 6.5–5.2 Ma^43^); 5.2 Ma early inception of La Palma (previous early estimate at 4.0 Ma^27–29^); 1.9 Ma inception of La Palma or La Palma Ridge (estimated 1.7–1.65 Ma^27–29^); and 1.1 Ma inception of El Hierro (estimated 1.12–0.88 Ma^29^).

Beds older than 17.0 Ma lack geochemical data to better constrain provenance. However, a short interval of events at 18.4 Ma may reflect the inception of Lanzarote (Fig. 4), which is supported by ages of 19.0 Ma obtained from the basal complex on the island^44^. The inception of Fuerteventura is more difficult to unravel. Pale-grey beds dated at 20.5–19.6 Ma coincide with earliest volcanism in the Southern Volcanic Complex, whilst beds dated between 23.1 and 21.0 Ma coincide with earliest volcanism in the Northern and Central Volcanic Complexes (Fig. 4). However, a series of pale-grey beds are dated between 24.7 and 26.5 Ma that may represent the initial inception of Fuerteventura, as these dates align with the oldest Oligocene ages from the Basal Complex of the island (Fig. 4)^45^. Older dates from the Basal Complex exist but may be affected by metamorphism^29,43,44^.

The most voluminous pale-grey ‘non-volcanic’ beds notably occur in the post-7.0 Ma record and are specifically coincident with: subaerial emergence of Tenerife following inception of Teno massif at 7.0–7.3 Ma; inception of Anaga massif and subsequent subaerial growth of it and the Teno massif on Tenerife at 6.0–5.1 Ma; initiation of Roque Nublo subaerial edifice growth on Gran Canaria at ~4.3 Ma; inception of La Palma between 4.8 and 4.0 Ma; and inception of the La Palma Ridge and El Hierro island between 2.0 and 1.2 Ma (Fig. 6). As well as demarking the points of inception of volcanic islands, the deposition of thinner pale-grey ‘non-volcanic’ turbidites also occur coincident with periods of subaerial volcanism, including: subaerial emergence of La Gomera and/or second eruptive phase of Gran Canaria at ~13–11.5 Ma; subaerial growth on La Gomera associated with the Upper Old Series basalts and Roques del Conde massif on Tenerife at ~9.0 Ma; growth of the Cañadas edifice on Tenerife commencing at ~3.0 Ma; and finally most recent volcanic activity on La Palma and Tenerife (0.8–0.4 Ma) (Fig. 6).

### Preconditioning and triggering of seafloor failures

**Fig. 7:**
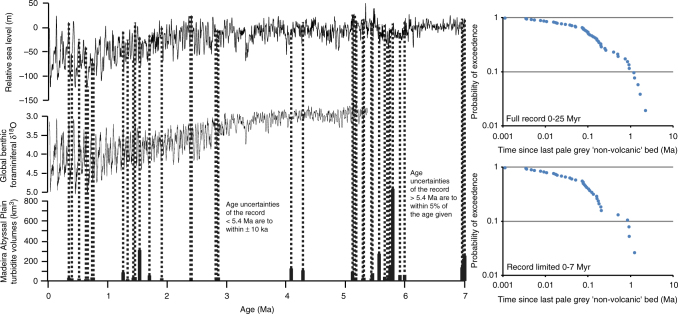
Comparison of the timing of pale-grey non-volcanic turbidites. Comparison of turbidite is since 7.0 Ma with a relative sea-level curve^76^. In addition, log–log plots of the probability of exceedance of the timing between turbidite events showing a near, but not perfect, exponential distribution defined by a series of linear distributions

The average recurrence of these the pale-grey ‘non-volcanic’ turbidites during the last 7.0 Ma is 0.22 Myr, which is much lower than 0.59 Myr for the period 17.0 to 7.0 Myr, and 0.52 Myr for the period older than 17.0 Ma. Probability of exceedance plots of the recurrence time between the pale-grey ‘non-volcanic’ turbidites show a series of three linear distributions of recurrences < 0.07 Myr, between 0.08 and 0.15 Ma and between 0.15 to 0.2 Ma, and a coarse tail of recurrence values between 0.3 and 1.1 Ma (Fig. 7). Exponential rather than linear distributions on log–log plots suggest the influence of a Poissonian, or otherwise random, control, whereas linearity indicates events of common recurrence that imply a non-random control (Fig. 7)^32,46^. These multiple linear distributions would imply a number of non-random processes potentially affecting the recurrence of these slides, rather than simply one cyclical process such as climate and associated sea-level change (Fig. 7).

We infer these different non-random distributions to reflect volcanic processes. Small recurrences of <0.07 Myr likely represent events clustered within a particular phase of volcanic growth on a particular island. The recurrences of the two distributions between 0.8 and 1.2 Myr may represent the timing between different phases of growth on the islands, with these two distributions reflecting recurrence between phases of submarine and subaerial growth, respectively. The recurrence values within the coarse tail are potentially a product of having records from seven different islands; these larger recurrence values may reflect the different timings of inception on the different islands.

The slides initiating the pale-grey ‘non-volcanic’ turbidites are shown to be concurrent with inception, ascension, emergence and subaerial growth of the Canary Islands (Figs. 4 and 6), thus volcanism and related seismicity likely ultimately trigger failure of the seafloor. Indeed, eruptions on Stromboli in 2002 caused subaerial flank collapse and generation of tsunamigenic landslides^37^, while seismicity linked to eruptions on the scale of caldera-collapse is suggested to be required for large-volume volcanic flank failure^47^. We demonstrate here that large volume seafloor failures occur during the early stages of island development and prior to protracted periods of subaerial volcanism and edifice growth on emergent islands. Landsliding during these two respective periods implicates the ascent of magma and igneous intrusion as an overarching process responsible for destabilising the submarine flanks^1,3–5^.

The Hurst exponent (*K*) for the complete record of events to 25 Ma is *K* = 0.52 (*N* = 67); whereby *K* = 0.5–0.6 indicates no significant trend-reinforcement. This implies that these landslides are likely randomly distributed temporally or non-clustered with respect to preceding or succeeding events^32,46^, and thus contradicts the results of the probability of exceedence plots. However, the Hurst exponent is most effective in datasets with *N* > 100 and furthermore cannot differentiate the input from numerous potentially non-random processes (identified above) from seven different island sources^48^; the Hurst exponent is thus considered unreliable in this situation.

Comparison of individual beds in the last 7.0 Myr to a relative sea-level curve reveals that 73% of slides (*N* = 37) occurred during rising or peak relative sea level, which contradicts the Hurst exponent further by indicating the influence of a further cyclical process on landslide recurrence (Fig. 7). This is supported whereby 80% of beds occur at the transition between dark brown or dark grey lowstand hemipelagites to light brown or white highstand hemipelagites, which demark the transition towards rising sea-level^49^. Subaerial volcanic island flank collapses have been implicated with environmental conditions at the transition from lowstand glacial to highstand interglacial^2,9,12,14^. Here, we suggest that while environmental conditions linked to sea level state, in particular sea-level rise, may precondition failure of these new submarine failures, ultimate triggering of the slides relies upon magmatic processes or associated seismicity.

Our study has implications for the geodynamics of ocean island archipelagos and the geohazards concurrent with their inception, ascension, emergence and subaerial growth. These early periods of island growth during a seamount-phase and then early subaerial emergence are synonymous with rapid growth and inflation of the submarine flanks by magma ascent and intrusive igneous activity^1,3–5^. Coincidence between subaerial flank collapse and volcanism has been inferred in the Canary Islands and at other volcanic islands, such as Montserrat or Stromboli^2,9,12–14,47,50–55^. However, failures during these early stages of island development in the Canary Islands, or indeed other island arc systems, have not been identified before. The implication is that early growth stages of volcanic islands are capable of instigating prodigious submarine failures equal or larger in volume than their subaerial counterparts, and thus pose significant geohazards. While subaerial flank collapses from active island edifices are potentially tsunamigenic^35,36,55,56^, there is also evidence that large landslides in greater water depths can also be tsunamigenic (e.g., 1946 Aleutians^57^, 1992 Central America^58^, and 1998 Papua New Guinea^59^ tsunamis). Therefore, although the wholly submarine failures in our study have an, as yet, un-quantified tsunamigenic capability, there are precedents for their potential to generate catastrophic tsunami; a further impetus for us to characterise their past occurrence, magnitude and preconditioning/trigger factors. An additional hazard posed is these large-volume slides may impact on volcanism by unloading and depressurising the magma chamber at depth^13,60^. Such large volume failures during the early stages of island development may therefore impact upon the subsequent volcanism and growth of the island.

### Multi-stage or single block failures

**Fig. 8:**
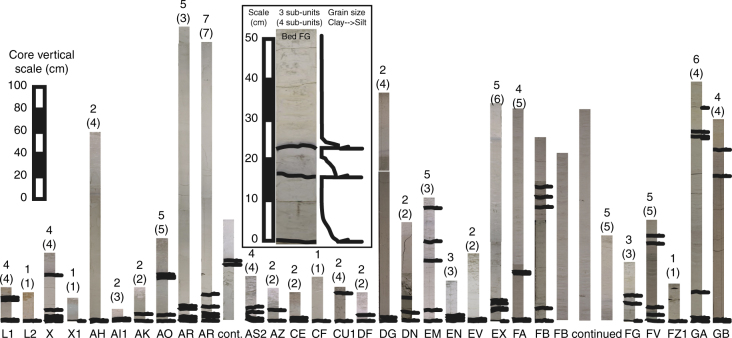
Summary of pale-grey non-volcanic turbidites in ODP Core 950 greater than 10 cm thick showing presence or absence of subunits. Base of sub-units highlighted on the turbidite photographs (black lines parallel to base). Number of sub-units present in the bed in ODP Site 950 is given above the maximum number of sub-units in brackets from the same bed in ODP Sites 951 and 952. Inset shows a close-up exemplar photograph of Bed FG with a corresponding grain-size profile. Sub-unit bases are arrowed

Determining whether these slides have occurred as single large-volume blocks or whether they are multi-stage and have occurred as closely spaced sequential series of failures from the same source location is important. This is because volume is a major determining factor in the potential size of a resulting tsunami from landslides^61^. Multi-stage failures that release the volume of the slide in a sequential series of smaller failures have reduced tsunamigenic potential^36,53,62^. Turbidite event beds can record whether the source slide was single or multi-stage by whether the deposit comprises one or multiple fining-upwards sequences called sub-units^53,62^. These distal deposits representing failures of the submarine flanks of the Canary Islands and adjacent seafloor predominantly comprise two or more silt layers in the basal portion of the deposits (85% of those beds thicker than 10 cm), which are separated by centimetres to decimetres of turbidite mud (Fig. 8). This implies that the slides were predominantly multi-stage, thus despite their large-volume, their tsunamigenic potential is reduced.

## Discussion

Here, we highlight a novel and potentially dangerous geohazard. Subaerial landslides, especially those in the Canary Islands, are easily recognised and mapped and have been well-dated often using a combination of independent methods resolving the ages of the onshore scar and the offshore deposits, e.g., Orotava slide on Tenerife^2,8,9,12–14^. However, the turbidites of the present study likely represent the only evidence of these large submarine landslides they represent, as the failure scars are likely infilled by thick accumulations of hemipelagites and subsequent proximal landslide deposits. Our study also highlights the minimum ages of inception for each of the Canary Islands. Dating of island inception has previously been completed by K-Ar dating of intrusion igneous bodies within exposed basement complexes commonly affected by metamorphism^25–29,39–41^. Our age estimates are consistent with published results (Figs. 4 and 6; Supplementary Table 1), but potentially offer refined estimates regarding the ages of island inception unaffected by metamorphism. Therefore the ages of inception and subsequent volcanic growth determined from mass wasting in our study provide invaluable insight into the geodynamics of ocean islands. We also show that although often large in volume, many of the slides that generated these deposits were multi-stage and likely occurred in a closely-spaced sequence of failures, rather than single block failures.

Our identification of potential submarine landslide hazards during early and continued island growth is critical, considering there are over 30 intraplate oceanic hotspots globally with islands and seamounts exhibiting volcanism. Determining the scale of this mass wasting is essential as submarine landslides on the scale of hundreds of cubic kilometres may be tsunamigenic, and thus may pose hazards capable of affecting entire ocean basins. However, also indicating that these landslides occur predominantly as multi-stage failures implies that their overall mass is divided among smaller events and thus may yield reduced tsunamigenesis.

## Methods

### Turbidite lithostratigraphy and geochemical compositions

The 17 Ma to present stratigraphy of the Madeira Abyssal Plain was constructed using ODP Sites 950, 951 and 952^2,9,38,63^. The stratigraphy from 43.0 to 18.0 Ma was constructed using only ODP Site 950, as it penetrated deeper into the stratigraphic record (Fig. 4). The first objective was to differentiate the pale-grey non-volcanic turbidites within the turbidite records, achieved by identification of their pale-grey colour and low magnetic susceptibility, which distinguishes them from volcaniclastic turbidites. Previous work has resolved the frequency of turbidites and the compositional differences between the types of turbidite^2,30^. Thus far, no studies have dated individual pale-grey non-volcanic turbidites, nor white calciclastic turbidites, nor the brown turbidites. The geochemical composition of these beds are derived from bulk geochemistry from ICP-AES and ICP-MS analyses, and comprises both published and unpublished major, trace and REE data^2,30,63^. The compositions of these beds may also reveal new insights into their provenance. The reliability of major element oxides > 2 wt% present standard deviations 0.7–1.5% of the value, while values 1–2 wt% have standard deviations 1.5–10% of the value. With regard to accuracy of wt% calculations for major element oxides in relation to international standard reference materials for accuracies are better than 2% of the value when > 3 wt%, while within 2–10% of the value when 0.1–2 wt%. Reliability of trace element data have standard deviations better than 2% with values > 60 ppm, while accuracy is within 0.7–5.0% of the value with values > 60 ppm.

### Landslide age-model

This study aims to accurately date the pale grey ‘non-volcanic’ turbidites to reconstruct the record of landslides from the seafloor surrounding the Canary Islands. The dated record of slides will provide a means to evaluate their triggering, by comparing to ages of the seamount phases of the Canary Islands and ages of volcanism on the islands. In the last 1.2 Ma individual turbidites can be dated with conservative estimated uncertainties of ±10 ka^9,49^. This is based upon the relative coccolith species abundances, combined with absolute dates of first and last coccolith occurrences, magnetostratigraphic markers, and lithostratigraphy of the hemipelagites sucession^2,9,49^. In the record between 1.2 and 5.4 Ma absolute dates of first and last coccolith occurrences and magnetostratigraphic markers provide dating accuracies to within 1–5% of the age, thus uncertainties initially between 12 and 270 ka^38^. However, up to 5.4 Ma the cycles of lowstand and highstand are defined by odd and even-number Marine Isotope Stages (MIS) that have been robustly determined^64^. Ages of individual turbidites are ascertained by interpolation of hempelagite sedimentation rates between datum horizons. For those beds in the last 5.4 Myr, these ages are examinated against the turbidite position in the hemipelagites lithostratigraphy and the inferred position within the marine isotope stage history.

Previous work has identified that the lithostratigraphy of the hemipelagite succession records periods of sea level change, manifested in the MIS record, because of the influence on bottom waters in the Madeira Abyssal Plain being sensitive to sea-level state^9,25,46,47,49^. Thus at high sea-level there is greater calcium carbonate preservation forming white calcareous oozes younger than 2.5 Ma, or pale brown to light grey hemipelagites in the older record. Conversely, dark brown clays were deposited during lowstands younger than 2.5 Ma and dark grey clays in the older record. The boundaries between these bipartite hemipelagite units are sharp and distinct, and mark the boundaries between highstand (odd numbered) and lowstand (even numbered) MIS periods^9,49,64^.

All the MIS stages are recorded in the hemipelagite sequences at ODP Sites 950, 951 and 952 up to 5.4 Ma (Fig. 5), apart from between 4.0 and 3.9 Ma and 4.75 and 4.2 Ma where the sea-level perturbations were not sufficient to influence bottoms waters enough to generate differences in hemipelagite lithology. Otherwise, a robust lithostratigraphy provides a conservative dating accuracy of ± 10 ka from 5.4 Ma until present for those turbidites at MIS boundaries (80%). Those beds located beyond a specific boundary (20%) are constrained by the dates of the MIS boundaries above and below and an age is extrapolated from its position relative to the MIS boundaries above and below that yield a dating accuracy less constrained at ± 20 ka. Older than 5.4 Ma the MIS boundaries are less well constrained. In the record between 5.4 and 6.5 Ma, coccolith biostratigraphy and magnetostratigraphy still provide dating accuracies of 1% (54–65 ka), while in the record older than 8.00 Ma this increases to accuracies only to within 5% on average^38^. This means that for those turbidites in the record older than 17 Ma the dating uncertainties may be as high as 0.8–2.2 Ma.

### Turbidite and landslide volumes

The volume of the deposits, and the slides by association, were derived using the methodology utilised by Hunt et al.^2^ First, due to the depth of burial of the older strata these volumes are decompacted to enable valid comparison between the strata^2^. The ODP Cores can be linked to a dense network of 2D seismic reflection lines via a seismic stratigraphy and a number of key reflectors (Supplementary Fig. 1)^65,66^. These seismic packages have been delineated across the Madeira Abyssal Plain providing volumes in situ^31,66^. To provide volumes on deposition the seismic unit volumes are also decompacted following^31,66^. To calculate turbidite volumes at the point of deposition, and slide volumes by proxy, the initial turbidite thickness is calculated by decompacting the entire stratigraphic sequence incrementally. The ratio of the decompacted thickness of the single turbidite to the decompacted thickness of its seismic unit is taken and reported as a function of the total decompacted volume of the seismic unit to give the decompacted volume of the single turbidite. This is repeated for each bed and repeated at each ODP Site to better understand the uncertainty in the volumes calculated, which are between 10 and 30%.

Turbidites deposited during the last 550 ka are correlated consistently across the Madeira Abyssal Plain^9,67^. Plots of turbidite bed thickness across the entire Madeira Abyssal Plain incorporating all available piston cores and ODP data show that the beds thicken into subtle basin lows and taper towards the basin fringes (Supplementary Fig. 2). Although there are some variations in how these beds thicken and thin, these differences contribute only 10–30% uncertainty in volume estimates across the basin. They generally pond consistently within basin lows and taper towards basin edges (Supplementary Fig. 2). Thus the relative thicknesses of the turbidites are generally consistent across the basin, validating the assumption that the thickness at the ODP Sites reflect their relative thickness across the basin. Piston core studies of the Madeira Abyssal Plain show than beds are distributed across the entire depocentre (Supplementary Fig. 2)^67^. Volume estimates generated from previous isopachs of individual beds incorporating variable lateral thickness from over one hundred piston cores produce results within 20% of the estimated volume from the ODP Cores taking an average thickness from the Sites 950, 951 and 952 and extrapolating across the basin. Seismic surveys implicate the potential that from 12 Ma onwards the basin was consistently and entirely flooded by turbidity currents, prior to 12 Ma there is reduced potential coverage of the entire basin by turbidity currents, which increases the uncertainty in volume estimates in those older beds^19^.

Flows are capable of eroding material along their pathway, thus the final volume of the turbidite may be up to 50% larger by accumulating that sediment^68^. Specific evidence of erosion beneath turbidity currents from volcanic island flank collapses indicates this value is less at 10%^53^. Thus far evidence investigating these large turbidity currents has shown that their volumes can be increased greater than this, thus their initial volumes are still on an order of >10 km^3^, and thus must remain sourced from submarine landslides, as pyroclastic flows, hypopycnal flows and delta front collapses cannot yield flows of these large magnitudes^9,32,68–70^. We also note that these volumes, whilst including eroded material, are likely the minimum volume estimate for the event, as we cannot resolve a total volume inclusive of the volume deposited as a proximal landslide or debris avalanche deposit^2,9,59^.

### Statistics of landslide recurrence

Statistical analysis of the recurrence of the pale-grey ‘non-volcanic’ beds followed the methods of Hunt et al.^2,49^ and Clare et al.^32,46^. The recurrence times of the beds were analysed on probability of exceedance plots. Probability of exceedance plots of the recurrence time between the pale-grey ‘non-volcanic’ turbidites may provide insights into type of process(s) controlling recurrence (Fig. 6). Exponential distributions on log–log plots suggest influence of a Poissonian, or otherwise random, control. Linear distributions of recurrence may indicate events of common recurrence and imply a non-random control^32,46^. The Hurst exponent (*K*) is used to identify clustering and whether there is trend-reinforcement. *K* = 0.5–0.6 indicates no significant trend-reinforcement, which implies a likely randomly or non-clustered distribution with respect to preceding or succeeding events, and suggests a control on the distribution is unlikely to be a cyclical or rhythmic process^32,46^.

Single volcaniclastic turbidite beds deposited in the Agadir Basin and Madeira Abyssal Plain linked to subaerial Canary Island landslides have been found to comprise multiple fining-upwards sequences known as sub-units^53,62^. This facies has been identified as representing the multi-stage failure mechanism of the source landslide^53,62^. Therefore where this facies is seen in the pale-grey ‘non-volcanic’ beds, this is evidence of potential multi-stage collapse. Inclusion criteria are that first, the beds have to be present in all three ODP Sites so that the continuity of having two or more sub-units can be assessed, and second, the beds are greater than 10 cm in thickness, as beds thinner than 10 cm pose difficulties in distinguishing the presence of single or multiple fine silt layers.

### Data availability

Geochemical data is available from Jarvis et al. (1998) (10.2973/odp.proc.sr.157.129.1998)^30^. Remaining data from this article is available from the corresponding author upon request.

## Electronic supplementary material

Supplementary Information
