# Supplementary material for: Prodigious submarine landslides during the inception and early growth of volcanic islands
Source: Nat Commun. 2017 Dec 12;8:2061. doi: 10.1038/s41467-017-02100-3 (PMC5727060; doi:10.1038/s41467-017-02100-3)
Supplement: Supplementary file 1 — Supplementary Information [file 41467_2017_2100_MOESM1_ESM.pdf]

Supplementary Table 1. Pale-grey 'non-volcanic' turbidite origin in the last 17.0 Ma.

Turbidite Group (numbering convention from Hunt *et al.* 2014)	Bed Ages (Ma)	Bed Volumes (km^3)	Chemical Composition	Prescribed Origin
JI to JM	15.7 to 16.55	15–85	Not determined	Volcanism on Fuerteventura or potential initiation of Lanzarote or La Gomera.[1]
JH	14.2	110	Not determined	Initiation of Gran Canaria.[1,2,3]
JE to JG	12.5 to 13.0	20–60	Not determined	Magma ascension associated with continued growth on Gran Canaria.[1,2,3]
JC and JD	11.5 to 11.75	20–70	Not determined	Possible later initiation of La Gomera.[1,3,4] Alternatively, early volcanism of the Roque de Conde shield on Tenerife at 11.8 to 10.9 Ma.[5,6]
GX, JA and JB	8.8 to 9.25	10–50	Not determined	Continued magma ascension associated with shield growth of La Gomera responsible for Upper Old Edifice 1 Eastern Felsic Rocks.[4,6] Although ages of Roque de Conde Massif on Tenerife show volcanism and growth continues there from 11.9 to 8.9 Ma.[5]
EN, EV, EV1, FA, FA1, FA2, FB, FG, FG1, FV, FZ1 and GB	5.17 to 5.91 and 6.93 to 7.15	20–105	High K/Al, high Fe/Al , moderate to high Ti/Al, lower Zr/Al and lower Cr/Al.	Emergence of Tenerife at ~7 Ma.[3] Magma ascension associated with growth of the Teno Massif on Tenerife at 5.99–6.11 Ma and 5.54–5.25 Ma.[5] Development of Anaga Massif 4.89 to 4.72 Ma.[5]
EM,EQ2, EQ3, EU2, EV1, EX, EZ2, GA and GA1	5.12 to 5.65 and 7.05 to 7.08	13–260	Low K/Al, low Ti/Al, low Fe/Al, low Mg/Al, high Cr/Al and high Zr/Al.	Composition different from early shied building growth on Tenerife. Ages prior to and coincidental with voluminous felsic growth of La Gomera during later Upper Old Edifice 2 Vallehermoso Felsic rocks (7.5–6.4 Ma) and Young Edifice 1 (5.7–4.6 Ma).[6]
DG and DN	4.10 to 4.28	90–115	High K/Al, low Fe/Al and moderate Ti/Al.	Renewed volcanic activity on Gran Canaria after a volcanic gap.[7] Composition different to events attributed to Tenerife.
CE and CF	2.84 to 2.85	10–25	Very high K/Al and moderate Fe/Al, while CE has low Ti/Al but CF has moderate Ti/Al.	CE and CF coincidental but different in composition. CE may be due to magma ascension and volcanism on Gran Canaria following period of quiescence.[7] Whilst CF is synonymous with development of Las Canadas on Tenerife.[8]
AZ, AS1, AO, AO1 and AK	1.35 to 1.94	15–25	High K/Al, low Ti/Al and moderate Fe/Al.	Low volume events coincidental with inception and early growth of La Palma from 2.0 to 1.46 Ma.[10]
AR	1.71	300	Marginally different from AZ, AO, AO1 and AK with moderate K/Al, low Ti/Al and moderate to high Fe/Al.	Age is similar to that of the earliest volcanism on La Palma and inferred to represent failure as result of inception of La Palma or La Palma Ridge at 1.77 Ma.[9]
AH	1.28	76	Distinct from previous beds AZ to AI. High low Fe/Al, low Ti/Al and low Ti/Al. Moderate La and Th.	Composition is different from previous events and the date is immediately prior to basement complex volcanism on El Hierro. Inferred to result from inception of El Hierro.[10]
X1, X2, S2, P1 and P2	0.65 to 0.77	5.5–25	Low Ti/Al, moderate to high Fe/Al and moderate K/Al	Coeval with 0.71 and 0.65 Ma volcanism marking southward extension of the Taburiente volcano towards the Palma ridge south of the island. [11] Composition is similar to those from Tenerife and most likely marks period prior to extensive explosive volcanism of the Las Canadas edifice.[9]
L2, L3 and N1	0.35 to 0.49	12–20	Low Ti/Al, moderate to high Fe/Al and moderate K/Al	Compositions differ from X1 and X2, but similar to AZ, AO and AK. Potentially associated with magma ascension related to Cumbre Vieja eruptions on La Palma.

[1]Abdel-Momen *et al.* 1971; [2]McDougall and Schmincke, 1976; [3]Carracedo *et al.* 1999; [4]Paris *et al.* 2005; [5]Thirwall *et al*. 2004; [6]Anchochea *et al*. 2006; [7]Guillou *et al*. 2004; [8]Abdel-Momen *et al.* 1972; [9]Haugen *et al.*, 2005; [10]Carracedo *et al.* 2001; [11]Ancochea *et al.* 1994.

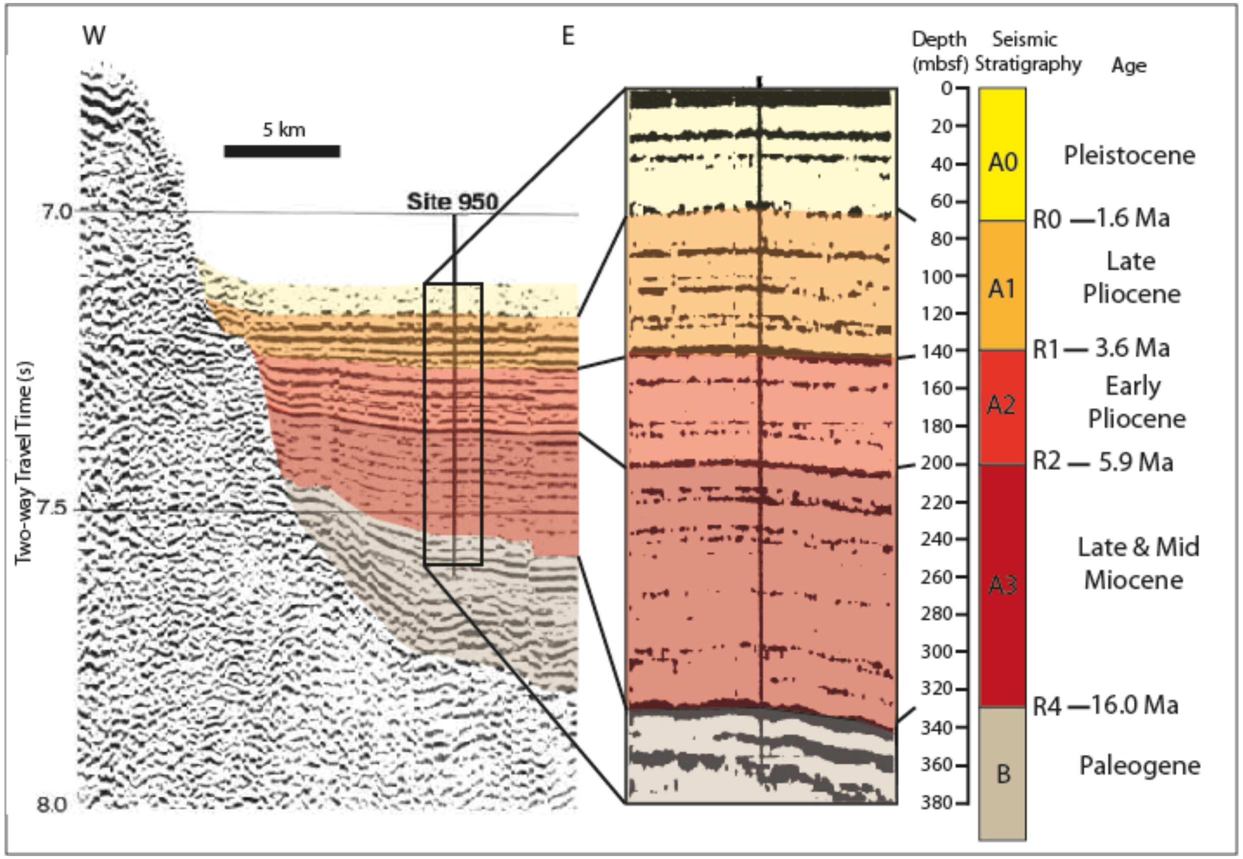

Supplementary Figure 1 | Seismic reflection profile at ODP Site 950 showing seismic stratigraphy correlated with the core stratigraphy, highlighting the presence of five seismic intervals. These seismic intervals have previously been mapped across the Madeira Abyssal Plain to provide volume estimates that have been subsequently decompacted.

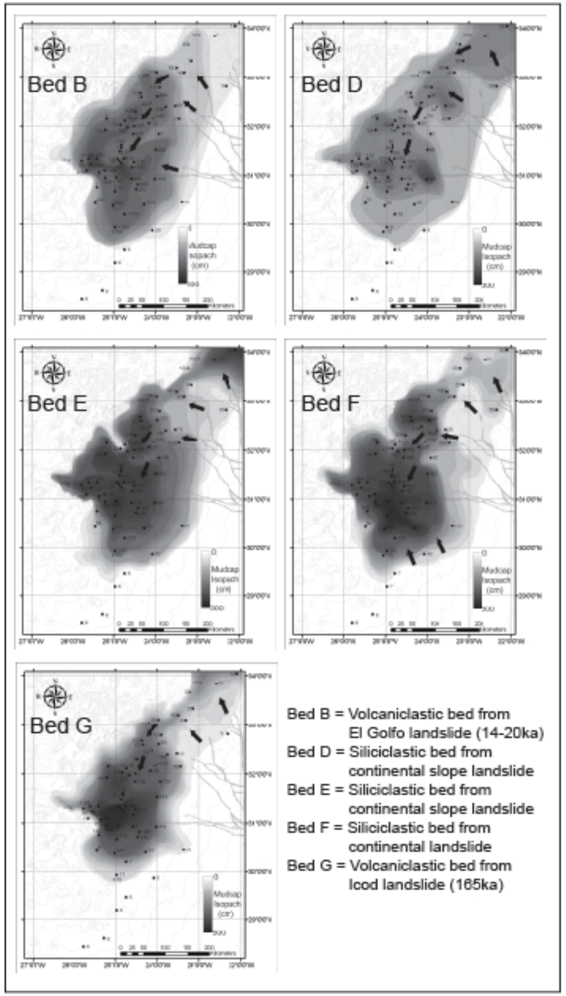

Supplementary Figure 2 | Turbidite isopach maps of the last five large-volume turbidites in the Madeira Abyssal Plain based upon over 300 piston cores. These include beds B and G representing slides from El Hierro and Tenerife, respectively.

Supplementary References

1. Abdel-Monem, A. et al. Potassium-argon ages, volcanic stratigraphy, and geomagnetic polarity history of the Canary Islands: Lanzarote, Fuerteventura, Gran Canaria, and La Gomera. *Am. J. Sci.* **271**, 490–521 (1971).
2. McDougall, I. and Schmincke, H.U. Geochronology of Gran Canaria, Canary Islands: age of shield building volcanism and other magmatic phases. *Bulletin Volcanologique* **40**, 1, 57-77 (1976).
3. Carracedo, J. C. Growth, structure, instability and collapse of Canarian volcanoes and comparisons with Hawaiian volcanoes. *J. Volcanol. Geotherm. Res.* **94**, 1–19 (1999).
4. Paris, R., Guillou, H., Carracedo, J. C. & Torrado, F. P. Volcanic and morphological evolution of La Gomera (Canary Islands), based on new K-Ar ages and magnetic stratigraphy: implications for oceanic island evolution. *J. Geol. Soc.* **162**, 3, 501–512 (2005).
5. Thirwall, M. F., Singer, B. S. & Marriner, G. F. 39Ar-40Ar ages and geochemistry of the basaltic shield stage of Tenerife, Canary Islands, Spain. *J. Volcanol. Geotherm. Res.* **103**, 1, 247–297 (2000).
6. Ancochea, E. et al. A new chronostratigraphical and evolutionary model for La Gomera: implicatiosn for the overall evolution of the Canarian Archipelgo. *J. Volcanol. Geotherm. Res.* **157**, 4, 271–293 (2006).
7. Guillou, Hervé, et al. The Plio–Quaternary volcanic evolution of Gran Canaria based on new K–Ar ages and magnetostratigraphy. *J. Volcanol. Geotherm. Res.* **135**, 3, 221-246 (2004).
8. Abdel-Monem, A., Watkins, N.D., & Gast, P.W. Potassium–argon ages, volcanic stratigraphy, and geo- magnetic polarity history of the Canary Islands: Tenerife, La Palma, and Hierro. *Am. J. Sci.* **272**, 805–25 (1972).
9. Haugen, K. B., Løvholt, F., & Harbitz, C.B. Fundamental mechanisms for tsunami generation by submarine flows in idealised geometries. *Mar. Pet. Geol.* **22**, 209–217 (2005).
10. Carracedo, J.C. et al. Geology and volcanology of La Palma and El Hierro, Western Canary Islands. *Estudios Geol*. **57**, 175-273 (2001).
11. Ancochea, E., *et al.* Constructive and destructive episodes in the building of a young oceanic island, La Palma, Canary Islands, and genesis of the Caldera de Taburiente. *J. Volcanol. Geotherm. Res.* **60**, 3, 243–262 (1994).
